# Comparative performance of COVID-19 serology testing

**DOI:** 10.1016/j.plabm.2022.e00289

**Published:** 2022-07-06

**Authors:** Nam K. Tran, Larissa May, Stuart H. Cohen, John Rodrigo, Raymond Gong, Ying Liu, Peter Conner

**Affiliations:** a Dept. of Pathology and Laboratory Medicine, UC Davis, United States; b Department of Emergency Medicine, UC Davis, United States; c Department of Internal Medicine, Division of Infectious Diseases, United States

**Keywords:** Antibodies, Hospitalization, Neutralization, Nucleocapsid, SARS-CoV-2, Spike, Titer, Vaccines

## Abstract

**Background:**

The 2019 novel coronavirus infectious disease (COVID-19) pandemic resulted in a surge of assays aimed at detecting severe acute respiratory syndrome (SARS) – coronavirus (CoV) – 2 infection and prior exposure. Although both molecular and antigen testing have clearly defined uses, the utility of serology remains uncertain and is presently not recommended for assessing immunity.

**Methods:**

We conducted a pragmatic, observational study evaluating four commercially available emergency use authorized laboratory-based COVID-19 serology assays (Assays A–D). Remnant samples from hospitalized, and non-hospitalized SARS-CoV-2 PCR positive patients, as well as vaccinated and unvaccinated individuals were collected and tested. Positive percent agreement (PPA) and negative percent agreement (NPA) were calculated. Antibody concentrations were compared across the platforms and populations.

**Results:**

A total of 588 remnant samples derived from 500 patients were tested. PPA at 5–12 weeks post-PCR positive results for Assays A-D was 98.3, 97.4, 99.2, and 95.8% respectively. NPA was 100% across all platforms. Mean antibody concentrations at 2–4 weeks post-PCR positive result were significantly higher in hospitalized versus non-hospitalized patients, respectively, for Assay A (131.8 [101.7] vs. 95.6 [100.3] AU/mL, P < 0.001), B (61.7 [62.4] vs. 38.1 [40.5] AU/mL, P < 0.001), and C (157.6 [105.3] vs. 133.3 [100.7] AU/mL, P < 0.001). For individuals receiving two vaccine doses mean antibody concentrations were respectively 169.6 (104.4), 27.3 (50.8), 189.6 (120.9), 21.19 (13.1) AU/mL for Assays A-D.

**Conclusions:**

Overall, PPA and NPA differed across the four assays. Assays A and C produced higher PPA and NPA and detected larger concentrations of antibodies following vaccination.

## 1 Background

The 2019 novel coronavirus infectious disease (COVID-19) pandemic has resulted in a surge of *in vitro* diagnostic (IVD) assays and platforms aimed at detecting severe acute respiratory syndrome (SARS) – coronavirus (CoV) – 2 [1]. Molecular- and antigen-based techniques identify active SARS-CoV-2 infection, while serology assays detect antibodies produced against the virus following infection or vaccination [2,3]. Although molecular and antigen testing have clearly defined roles, the utility of serology remains uncertain and is presently not recommended for assessing immunity in many countries [4].

From January 2020 to September 2021, 235 molecular, 88 serology/adaptive immunity, and 34 antigen have received United States Food and Drug Administration emergency use authorization (EUA) [5]. Of these 502, 85 are serology tests. These serology assays target either total antibodies (IgA, IgM, IgG), IgM/IgG, or IgG against either the SARS-CoV-2 nucleocapsid (N) or spike (S) proteins. Early serology assays were qualitative, with newer assays exhibiting linear performance for semi-quantitative or quantitative measurements – raising the possibility of evaluating antibody titers among individuals experiencing vaccine breakthrough infections and determining the need for vaccine booster shots [6,7].

Unfortunately, the sheer diversity of EUA serology methodologies and lack of standardization creates significant challenges for healthcare professionals [8,9]. Comparative studies are needed to aid clinical laboratories and infectious disease experts to determine which assays may better suited to address evolving pandemic demands. To this end, the objective of our study was to evaluate the clinical performance of EUA serology assays in vaccinated and previously infected non-vaccinated patients.

## 2 Methods

We conducted a pragmatic observational study evaluating commercial EUA laboratory-based COVID-19 serology assays available at our institution.

*Study Samples and Data:* Remnant serum samples from 500 adult (age ≥18 years) patients were aliquoted and frozen at −70 °C for batched serology testing. Samples were stored in our institution's College of American Pathologists (CAP) accredited biorepository. Patient demographics (*i.e.,* age and gender), vaccination status, number and timing vaccine dose, and number of days post molecular COVID-19 result. COVID-19 vaccines available at the time of the study were mRNA based (*i.e.,* Pfizer and Moderna) and require at least two doses to achieve maximum efficacy [10].

*Molecular Testing:* Molecular testing was performed as part of routine clinical evaluation of patients using EUA reverse transcription polymerase chain reaction (RT-PCR) SARS-CoV-2 assays. At our institution, two EUA RT-PCR assays were employed to support routine patient care needs: (a) laboratory-based high throughput batched testing platform (Cobas 6800, Roche Molecular Systems, Pleasanton, CA), and (b), a rapid point-of-care (POC) testing platform (Cobas Liat, Roche Molecular Systems, Pleasanton, CA). The high throughput RT-PCR platform targets the open reading frame (ORF) 1a/1b, and the envelope (E) protein regions within the SARS-CoV-2 genome. In contrast, the POC assay targets ORF1a/1b and N protein genes [11].

**Fig. 1:**
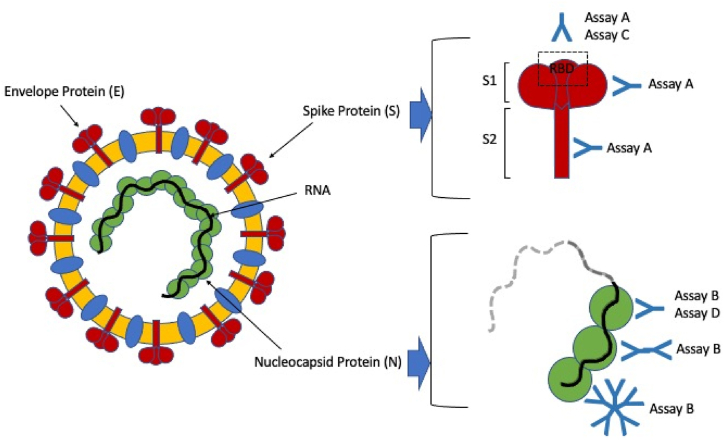
**Study Assay Serology Targets.** The figure provides a conceptual drawing of SARS-CoV-2 and enlarged illustrations of the spike (S) and nucleocapsid (N) proteins. Targets for each Assay are shown with Assay A targeting IgG antibodies against S1/S2 including the receptor binding domain (RBD). Assay B targets IgG, IgA, and IgM antibodies against N proteins. Assay C targets anti-RBD IgG, and Assay D targets anti-N IgG.

*COVID-19 Serology Assays:* Four EUA laboratory-based serology assays were compared in this study. Platforms were selected purely due to being available at our institution. Table 1 summarizes each assays methodology and anticipated performance for each method [12]. Fig. 1 illustrates antibody targets for each assay. Briefly, Assays A, B, and C were qualitative tests. Assay A (DiaSorin, Stillwater, MN) targeted IgG's against S1/S2 domains of the S protein. For Assay B (Roche Diagnostics, Indianapolis, IN), total anti-N antibodies (IgA, IgM, and IgG) were detected. Assay C (Roche Diagnostics, Indianapolis, IN) was the sole assay (anti-S IgG) evaluated in this study that was approved for semi-quantitative analysis. Lastly, Assay D (Abbott Laboratories, Abbott Park, IL) targeted anti-N IgG. It must be noted that although Assays A, B, and D were emergency use authorized as qualitative tests, these platforms also generate quantitative values reported as arbitrary units per milliliter (AU/mL). These quantitative values were used in this study to compare relative antibody titers across assays.

**Table 1:** Anticipated clinical performance of serology assays A – D.

Assay	Manufacturer	Targets	Methodology	PPA(%)	NPA(%)
A	Diasorin	Anti-S1/S2 IgG^a^	CLIA	97.6	99.3
B	Roche	Anti-N Total Ig^b^	ECLIA	100	99.8
C	Roche	Anti-S Total IgG^c^	Semi-Quant ECLIA	96.6	100
D	Abbott	Anti-N IgG	CMIA	100	99.0

**Abbreviations:** CLIA, chemiluminescent immunoassay; CMIA, chemiluminescent microparticle immunoassay; ECLIA, electrochemiluminescent immunoassay; Ig, immunoglobulin; N, nucleoprotein; NPA, negative percent agreement; PPA, positive percent agreement; S, spike protein.

Notes: ^a^S1 subunit of the spike protein contains the receptor binding domain; ^b^detects IgA, IgG, and IgM; ^c^includes antibodies against receptor binding domain of the spike protein.

*Statistical Analysis:* Positive percent agreement (PPA) and negative percent agreement (NPA) calculated for each assay at <2, 2–4 weeks, and 5–12 weeks post SARS-CoV-2 infection or vaccination. The use of PPA and NPA were used as surrogates for sensitivity and specificity respectively due to lack of a “gold standard” [13]. Mean antibody levels for each assay were compared between methods for each time window, as well as among patients who were vaccinated versus naturally infected. Antibody levels among naturally infected hospitalized and non-hospitalized patients were also compared.

## 3 Results

A total of 583 remnant serum samples from 500 adult patients were used for the study. Of the 500 patients, 120 were vaccinated individuals that had serology testing performed <2 weeks after the first vaccine dose. One-hundred and eight patients out of this 120 had serology testing at 2–4 weeks following the second vaccine dose. Additionally, among these 120 vaccinated individuals, 27 reported experiencing PCR confirmed COVID-19 before their first (n = 21) or second vaccine dose (n = 6). Eighty-three vaccinated patients had samples collected at <2 weeks following the first dose, and 2–4 weeks following the second dose.

Table 2 summarizes study patient demographics. At <2 weeks post-PCR result (n = 220 patients), PPA was 86.3, 86.2, 86.4, and 83.2% for Assays A-D respectively. Negative percent agreement for Assays A-D was respectively, 97.4, 94.3, 95.6, and 94.2%. When tested within 2–4 weeks post-PCR positive result (n = 72 patients), PPA was respectively 94.1, 94.1, 94.1, and 92.3% for Assays A-D, while NPA was 100% for Assays A-C, and 93.2% for Assay D. Positive percent agreement at 5–12 weeks (n = 199 patients) for Assays A-D was 98.3, 97.4, 99.2, and 95.8% respectively, while NPA was 100% across all platforms.

**Table 2:** Study sample demographics.

	Non-Vaccinated (n = 355)	Vaccinated (n = 145)^a^	P-Value
**Mean (SD) Age (Years)**	55.4 (19.9)	41.7 (12.9)	<0.001
*Hospitalized* (n = 143)	65.9 (14.9)	N/A	
*Non-Hospitalized (n = 212)*	52.9 (20.4)	N/A	
*Single Vaccine Dose (n = 120)*^*a*^	N/A	41.0 (10.2)	
*Two Vaccine Dose (n = 108)*^*a*^	N/A	42.6 (13.5)	
**Gender (M/F)**	212/143	81/147	<0.001
*Hospitalized*	50/93	N/A	
*Non-Hospitalized*	162/50	N/A	
*Single Vaccine Dose*^*a*^	N/A	45/75	
*Two Vaccine Dose*^*a*^	N/A	36/72	

**Abbreviations:** F, female; M, male; N/A, not applicable; and SD, standard deviation.

Note: ^**a**^Eighty three patients from this group had remnant samples after both first and second vaccine doses.

**Fig. 2:**
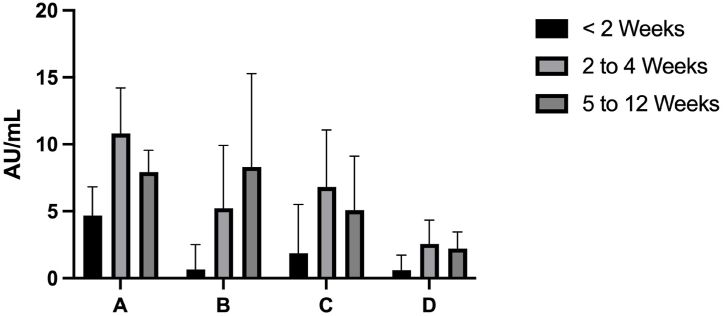
**Mean Quantitative Antibody Results Following COVID-19 Infection.** Mean antibody concentrations (y-axis) at <2 weeks (black bars) post-PCR positive result as reported by each assay (A–D) was 4.67 ± 2.15 (n = 112), 0.64 ± 1.87 (n = 114), 1.86 ± 3.64 (n = 109), and 0.59 ± 1.13 (n = 109) AU/mL respectively. At 2–4 weeks (light grey) post-PCR positive result 10.8 ± 3.41 (n = 16), 5.22 ± 4.71 (n = 16), 6.81 ± 4.27 (n = 16), and 2.55 ± 1.79 (n = 12) AU/mL for assays A-D respectively. For 5–12 weeks (dark grey) post-PCR positive results, mean antibody concentrations were found to be 7.93 ± 1.62 (n = 117), 8.31 ± 6.96 (n = 116), 5.07 ± 4.05 (n = 118), and 2.21 ± 1.24 (n = 114) AU/mL respectively. Standard deviations are indicated in the Figure by error bars.

**Fig. 3:**
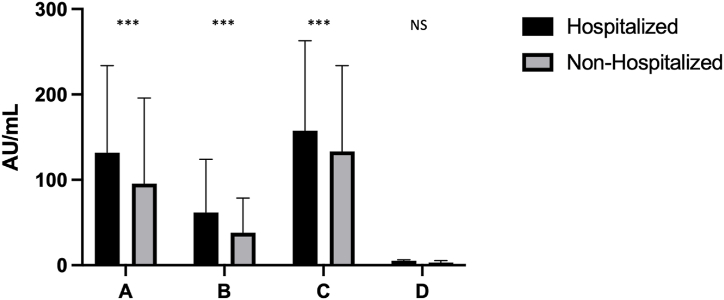
**Mean Quantitative Antibody Results Between Hospitalized and Non-Hospitalized Patients 2 to 4 Weeks Post-COVID-19 Infection.** Mean antibody concentrations (y-axis) at 2–4 weeks post-PCR positive result were significantly higher in hospitalized (n = 143) versus non-hospitalized non-vaccinated patients (n = 212), respectively, for Assay A (131.8 [101.7] vs. 95.6 [100.3] AU/mL, P < 0.001), B (61.7 [62.4] vs. 38.1 [40.5] AU/mL, P < 0.001), and C (157.6 [105.3] vs. 133.3 [100.7] AU/mL, P < 0.001). For Assay D, mean antibody concentrations were not significantly (NS) different between hospitalized versus non-hospitalized patients respectively (5.1 [2.2] vs. 3.1 [2.2] AU/mL, P = 0.852). Standard deviations are indicated in the Figure by error bars.

**Fig. 4:**
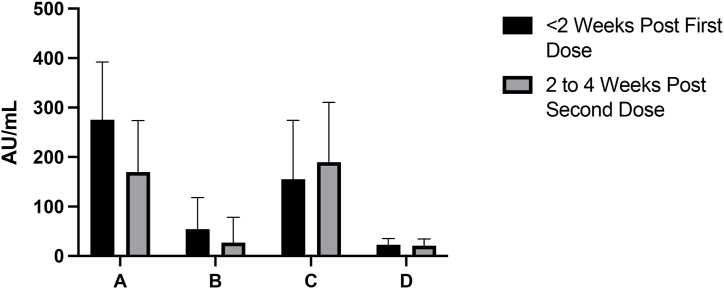
**Quantitative Antibody Results Following First and Second Vaccine Doses.** Mean antibody concentrations (y-axis) were 275.8 ± 116.1, 54.5 ± 63.6, 155.3 ± 119.0, and 22.7 ± 12.6 AU/mL for Assays A-D respectively when measured <2 weeks post-vaccination. Within 2–4 weeks post-vaccine dose (n = 108), mean antibody levels measured by Assays A-D were respectively 169.6 (104.4), 27.3 (50.8), 189.6 (120.9), 21.19 (13.1) AU/mL. Anti-N antibodies were not detectable for patients (n = 37) that were SARS-CoV-2 naïve. Standard deviations are indicated in the Figure by error bars.

Fig. 2 shows quantitative serology results in AU/mL for the four assays at <2 weeks, 2–4 weeks, and 5–12 weeks post-COVID-19 infection. SARS-CoV-2 antibody levels were also found to be significantly higher COVID-19 positive hospitalized compared to non-hospitalized patients when measured by the four assays (Fig. 3). Fig. 4 shows antibody levels <2 weeks following the initial vaccination dose, versus 2–4 weeks following the second vaccination dose, inclusive of the 27 patients that also reported having COVID-19 prior to completing the two-dose vaccine regimen.

## 4 Discussion

COVID-19 serology testing remains a controversial topic. Centers for Disease Control and Prevention interim guidelines for COVID-19 antibody testing notes that serology assays are not a replacement for virologic testing and such testing is currently not recommended to assess immunity [4]. The CDC also warns that serologic tests can vary in their individual performance characteristics. To this end, the goal of our study was to evaluate the performance of common laboratory-based EUA COVID-19 serology tests in PCR positive vs. PCR negative patients and further evaluate antibody levels in vaccinated versus unvaccinated individuals, as well as hospitalized versus non-hospitalized patients.

Assays A-C provide similar PPA at <2 weeks post PCR positive result, however only assay A achieved the highest NPA for this time window. At 2–4 weeks post-PCR positive result, Assays A-C performed similarly for both PPA and NPA. Interestingly, at 5–12 weeks post-PCR positive result, Assay C achieved the highest PPA, followed by Assay A. When comparing antibody levels, mean AU/mL were observed to be significantly higher among hospitalized versus non-hospitalized patients at 2–4 weeks post PCR-positive result when tested by Assays A-C. For vaccinated individuals, Assays A and C produced the highest quantitative values shortly (<2 weeks). At 2–4 weeks following the second vaccine dose, Assays A and C continued to report antibody levels at least ten times greater than Assays B and D – suggesting detected antibodies by these two assays may correlate more with vaccine response and perhaps immunity. Interestingly, only Assay C detected a rise in antibody levels compared to the other assays at 2–4 weeks following the second vaccine dose. This observation could be explained by poor standardization since some assays may detect anti-SARS-CoV-2 antibodies compared to others [14]. Detection of anti-N antibodies in vaccinated individuals were attributed to samples from the 27 subjects that were infected by SARS-CoV-2 prior to their first or second vaccine dose.

Both Assays A and C target anti-S IgG, with Assay A reporting strong correlation to neutralizing antibody tests [15], and Assay C being able to identify neutralizing antibodies in 72% of samples [16] in literature. Based on these specifications, it is plausible that these assays, both targeting anti-S IgGs, may be better suited for evaluating post-vaccine response. Performance of anti-S IgG for evaluating vaccine response has been recently illustrated. Bergwek et al., conducted a study evaluating antibody levels of COVID-19 vaccine breakthrough cases among healthcare workers in Israel [17]. Peri-infection anti-S IgG levels were lower in patients experiencing breakthrough cases compared to controls. It must be noted that the manufacturer for both Assay A and D have recently released semi-quantitative anti-S IgG assays which were not available at the time of this study [6,7].

Our study data highlights the need for better standardization for assay before defining antibody levels that correspond with protective immunity. Assuming standardization is possible, quantitative testing will be necessary, and thus, we will likely see a transition away from qualitative methods. Anti-S IgG assays are believed to better correlate with neutralizing antibodies and could be useful in evaluating response to S protein-based vaccines. However, anti-N antibodies should be undetectable in SARS-CoV-2 naïve individuals, so these assays may have clinical utility in identifying patients who have had a breakthrough infection [18].

Limitations of this study include being a single center evaluation and testing remnant samples based on clinical availability. This includes limiting our sample size from studying the effects of age on antibody levels which may differ between young versus older adults [19]. Additionally, only one platform (Assay C) in our study was approved for semi-quantitative results. Antibody titers from Assays A, B, and D were analyzed in this study to evaluate the relative difference between platforms. The study was intended to be pragmatic to capture as much real-world evidence to support laboratory decision-making when selecting COVID-19 serology tests for identifying prior SARS-CoV-2 exposure versus assessing potential immunity. This study was also conducted prior to the approval of vaccine booster doses (*i.e.,* third or fourth mRNA vaccine doses).

## 5 Conclusions

COVID-19 serology testing remains unstandardized with numerous platforms receiving FDA EUA since February 2020. The use of serology testing remains confounded by lack of real-world evidence identifying assays that provide optimal PPA and NPA for patients with prior exposure to SARS-CoV-2, as well as those receiving their first or second doses of mRNA vaccines. Our study found anti-S IgG (Assays A and C) to provide acceptable PPA and NPA for these roles and had means to continue detecting high levels of antibodies following vaccination. Further controlled studies are needed to define best practices for COVID-19 serology testing especially with the wider adoption of quantitative and semi-quantitative assays.
